# Evaluation of Energy Balance Estimated From Total Energy Expenditure and Body Composition Changes in Junior Sumo Wrestlers: An Observational Study Over Six Months

**DOI:** 10.7759/cureus.61158

**Published:** 2024-05-27

**Authors:** Miori Ogawa, Akiko Uchizawa, Shinsuke Tamai, Reiko Momma, Daisuke Hoshi, Emi Kondo, Hiroyuki Sagayama, Koichi Watanabe

**Affiliations:** 1 Institutes of Health and Sports Sciences, University of Tsukuba, Tsukuba, JPN; 2 Pediatrics, Japanese Red Cross Nasu Hospital, Otawara, JPN; 3 Research, Japan Society for the Promotion of Science, Tokyo, JPN; 4 Department of Sports Science and Research, Japan Institute of Sports Sciences, Tokyo, JPN; 5 Human Informatics and Interaction Research Institute, National Institute of Advanced Industrial Science and Technology, Tsukuba, JPN; 6 Department of Health and Sport Sciences, Osaka University of Health and Sport Sciences, Osaka, JPN

**Keywords:** obesity, bioelectrical impedance analysis, doubly labeled water, sumo wrestler, overweight athlete

## Abstract

Background

Sumo is a sport that requires wrestlers to develop their physique from childhood for athletic advantage. However, the energy expenditure and energy balance required for the growth of junior Sumo wrestlers remain unclear. This study aimed to determine the energy balance of junior Sumo wrestlers over six months using doubly labeled water (DLW) and bioelectrical impedance analysis (BIA).

Methodology

A total of 12 male Sumo wrestlers were affiliated with a local Sumo club (average age = 15 ± 1 years). The total energy expenditure (TEE) was measured using DLW, whereas body composition was evaluated using BIA. Daily physical activity was quantified using a tri-accelerometer (Active style Pro HJA-750C).

Results

The TEE was 4,194 ± 734 kcal/day, while daily physical activity without training was 786 ± 50 minutes. Within six months, the body weight increased by 2.0 ± 3.2 kg, fat-free mass (FFM) was augmented by 2.1 kg, while fat mass did not change significantly. The surplus energy accumulated was 5.6 ± 213 kcal/day.

Conclusions

The excess energy of junior Sumo wrestlers predominantly increases their FFM. To increase their physical prowess, wrestlers adhere to a lifestyle characterized by high-intensity training and attenuated daily physical activity.

## Introduction

Sumo wrestlers maintain a large body size to gain a competitive advantage [1]. The sport is conducted within a 4.55-m ring, and being a heavier wrestler decreases the chances of being thrown out [2,3]. Both college and professional wrestlers exhibit high resting energy expenditure due to their large organ-tissue mass [4,5]. However, no study has evaluated the energy balance of growing junior Sumo wrestlers. An estimation formula for each age can calculate the energy requirements of a typical child; hence, it is necessary to study the energy metabolism and requirements of junior Sumo wrestlers.

The doubly labeled water (DLW) method is considered the gold standard for measuring total energy expenditure (TEE) because of its accuracy and noninvasive nature, as it simply involves collecting urine samples [6]. Bioelectrical impedance analysis (BIA) is inexpensive, easy to perform, portable, and useful for constant body composition measurement without radiation exposure or invasion [7]. Therefore, this study aimed to use DLW and BIA to evaluate the energy balance of growing junior Sumo wrestlers in terms of changes in body composition over six months.

## Materials and methods

Study participants

This study enrolled 12 male Sumo wrestlers (aged 13-17 years) from a local Sumo club that included nine designated certified prefectural athletes at the proficiency level required to participate in a national tournament in Japan. The study protocols were explained to all participants, and written informed consent was obtained before the commencement of the study. All procedures in this study were performed following the tenets of the Declaration of Helsinki. This study was approved by the Institutional Review Board of the University of Tsukuba, Institutes of Health and Sports Sciences (approval number: Tai 019-156).

This observational study had a follow-up duration of six months. The participants’ physical parameters were evaluated, and urine samples were collected at the Sumo Training Center. Body composition was measured using the DLW method at the beginning of the training period (baseline). Body composition was measured twice using BIA, in November and May, at six-month intervals.

Body size

The height of the study participants was measured using a stadiometer (DST-210S; Muratec-KDS Corp., Kyoto, Japan). The participants’ body weight was measured while wearing light clothing using a digital scale (MC780A-N; Tanita, Tokyo, Japan).

Body composition and total energy expenditure

The stable isotope of DLW (^2^H_2_^18^O) was administered orally, and the periodic collection of urine samples facilitated the analysis of the concentration of each stable isotope of deuterium (^2^H) and oxygen-18 (^18^O) to calculate energy expenditure. Before day zero, participants were instructed to fast after eating dinner and were only allowed to consume water until bedtime.

The amounts of ^2^H and ^18^O were determined using the estimated total body water (TBW) based on body mass × 0.6 before the start of the investigation. The dose volume was approximately 0.12 g/estimated TBW (kg) (99.9 atom% ^2^H_2_O, Taiyo Nippon Sanso, Japan) and approximately 1.8 g/estimated TBW (kg) (10.0 atom% H_2_^18^O, Taiyo Nippon Sanso, Japan). TBW was calculated based on the isotope ratios of deuterium and ^18^O in the sample. Additionally, urine samples were collected on day zero baseline, after dose at 12 hours, the morning of days one and three, the evening of day five, and both the morning and evening of day seven. All samples were stored at temperatures ranging between −30°C and −5°C in internally threaded polypropylene vials with a screw cap featuring a special silicone gasket for optimal sealing. The vials were wrapped tightly in Parafilm M (Bemis Co. Inc., Oshkosh, WI, USA). An isotope ratio mass spectrometer (Sercon Isotope Ratio Mass Spectrometers, CF 20-20; Sercon Ltd, Crewe, UK) was used to analyze the isotope concentrations in the urine samples. The isotope ratios of deuterium and ^18^O in the samples were used to determine the emission of stable isotopes during a specified period and acquire the values for carbon dioxide production, TBW, and TEE [8,9].

Body composition was assessed at baseline and after six months using BIA (MC-780A-N Tanita K.K., Tokyo, Japan). The relationship between baseline TBW, determined using DLW, and BIA-based TBW was expressed using the equation y = 1.0715x + 2.125, with an R^2^ value of 0.7398, indicating a significant relationship. Based on the obtained regression equation, the BIA-based TBW at six months was adjusted to obtain the adjusted TBW. The fat-free mass (FFM) at six months was derived from the adjusted TBW. Water content within the FFM for each age group was determined. Fat mass (FM) at six months was determined by subtracting the weight of the FFM from the total weight.

Calculation of energy balance

The energy accumulation of FM and FFM was ascertained based on alterations in body composition within six months. By considering the energy density of FM as 9.5 kcal/g and FFM as 1.0 kcal/g, the daily energy balance was deduced from the energy accumulation observed during the aforementioned six-month duration by employing the following formula [10]:

$EB (\text{kcal/d}) = 1.0 \times \frac{\Delta \text{FFM}}{\Delta t} + 9.5 \times \frac{\Delta \text{FM}}{\Delta t}$

⊿t = 221 days

Physical activity and heart rate

A tri-accelerometer was used to quantify the non-training daily life activities. A triaxial accelerometer (Active style Pro HJA-750C; OMRON Healthcare, Japan) was affixed to the waists of the participants to measure the amount of daily life activity other than bathing and Sumo training. Data were converted to metabolic equivalents (METs) using an Omron HJA-750C data collection software version 2.2. Then, METs were calculated for each activity. The physical activity intensity thresholds were defined as follows: sedentary behavior (SB, ≤1.5 METs), light physical activity (LPA, 1.5 to <3.0 METs), and moderate-to-vigorous physical activity (MVPA, ≤3.0 METs) [11].

At rest before the Sumo training, the Polar OH1 optical heart rate (HR) sensor (Polar Electro, Kempele, Finland) was attached to a band, strapped securely to the inner side of the lower leg, and taped to prevent it from shifting [12]. The HR data were collected during the entire Sumo training and stored in the devices’ internal memory. The data were uploaded to the Polar Flow web service (Polar Electro Oy) [13,14].

Statistical analysis

Data were analyzed and the results are presented as mean ± standard deviation (SD). Differences in body size and composition reference values were evaluated using the t-test. Linear regression analysis was used to assess the relationship between TEE and FFM. Body composition was measured using the DLW and BIA methods. Statistical analyses were performed using SPSS Statistics for Windows (version 23.0; IBM Corp., Armonk, NY, USA). A p-value <0.05 was considered significant.

## Results

Changes in body size and composition

The physical attributes of the participants are listed in Table 1. Within six months, a height increase of 0.8 cm and a weight gain of 2.0 kg were observed (height: pre 171.1 ± 3.8 vs. post 171.9 ± 4.2 cm, p = 0.045; body weight: pre 100.1 ± 18.0 vs. post 102.1 ± 19.6 kg, p = 0.049). However, no significant alterations were observed in body mass index (BMI), BMIz, or obesity rate.

**Table 1:** Characteristics of the study participants at baseline and after six months. n = 12; *: p < 0.001. Data were analyzed using paired-sample t-test; ^a^: Body mass index (BMI) was calculated using the participants’ height (cm) and weight (kg); ^b^: BMIz was calculated using the average and standard deviation of the BMI stratified by age, according to the guidelines of the Japanese Society for Pediatric Endocrinology; ^c^: Obesity rate (%): ((measured weight – standard weight)/standard weight) × 100. The degree of obesity was categorized as mild (20-30%), moderate (30-50%), and highly obese (>50%); ​​​​​​​^d^: Total body water (TBW) obtained using BIA was determined using the following equation using doubly labeled water (DLW): (y = 1.0715x + 2.125);​​​​​​​^e^: Fat-free mass (FFM) derived from total body water; ​​​​​​​^f^: Fat mass (FM) calculated by subtracting FFM from body weight; ​​​​​​​^g^: Using Wilcoxon signed-rang sum test.

	Baseline, mean ± SD	After six months, mean ± SD	Amount of change	P-value
Age (year)	15.1 ± 1.4	15.7 ± 1.4	0.6 ± 0.5	0.002
Height (cm)	171.1 ± 3.8	171.9 ± 4.2	0.8 ± 1.2	0.049
Weight (kg)	100.1 ± 18.0	102.1 ± 19.6	2.0 ± 3.2	0.045
BMI^a^	34.2 ± 5.8	34.5 ± 6.3	0.3 ± 1.3	0.355
BMIz^b^	2.4 ± 0.6	2.4 ± 0.7	0.0± 0.1	0.638^g^
Obesity rate (%)^c^	66.9 ± 27.3	67.8 ± 29.4	0.9 ± 6.2	0.641
%Fat	33.0 ± 5.3	31.8 ± 7.5	−1.2 ± 4.1	0.001^*^
TBW (kg)^d^	49.2 ± 6.3	50.8 ± 6.0	1.6 ± 2.5	0.048
FFM (kg)^e^	66.3 ± 8.5	68.4 ± 7.9	2.1 ± 3.3	0.049
FM (kg)^f^	33.8 ± 10.2	33.7 ± 12.9	−0.1 ± 4.8	0.940

**Figure 1:**
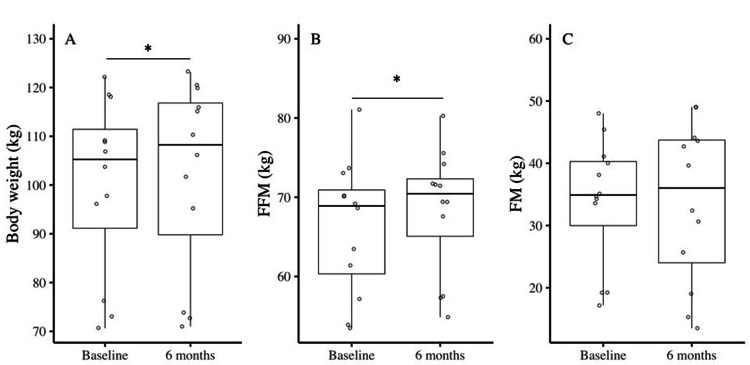
Changes in weight and body composition within six months in junior Sumo wrestlers. A. Body weight. B. Fat-free mass (FFM). C. Fat mass (FM). *: Significant difference at approximately six months (p < 0.05).

In the body composition, the TBW and FFM significantly increased within six months, while the percentage of body fat (%fat) and FM remained unchanged after six months (TBW: pre 49.2 ± 6.3 vs. post 50.8 ± 6.0 kg, p = 0.048; FFM: pre 66.3 ± 8.5 vs. post 68.4 ± 7.9 kg, p = 0.049; FM: pre 33.8 ± 10.2 vs. post 33.7 ± 12.9 kg, p = 0.940; %fat: pre 33.0 ± 5.3 vs. post 31.8 ± 7.5 kg, p = 0.323). Figure 1 illustrates the changes in body weight and composition.

Energy balance

The baseline TEE measured using the DLW method was 4,194 ± 734 kcal/day. The energy accumulation derived from the changes in body composition within six months is listed in Table 2. The total energy accumulation within six months was 1,132 ± 42,737 kcal/221 days, resulting in surplus energy of 5.6 ± 213 kcal/day.

**Table 2:** Metabolism and physical activity of junior Sumo wrestlers. n = 12; ^a^: Energy store of fat-free mass (FFM) was calculated as follows: (adjusted FFM measured by bioelectrical impedance (BIA) after six months) – (baseline FFM measured using the doubly labeled water (DLW) method). The adjusted FFM was obtained from the following relational expression using the baseline FFM and FM: y = 1.0715x + 2.125, R^2^ = 0.7398; ​​​​​​​^b^: Energy store of fat mass (FM) was calculated as follows: (weight after six months – adjusted FFM) – (baseline FM measured using the DLW method); ​​​​​​​^c^: Total energy accumulation for six months was obtained by summing the energy stores of FFM and FM; ​​​​​​​^d^: Total energy accumulation/kg for six months was obtained by dividing the total energy accumulation for six months by the weight change for six months; ​​​​​​​^e^: Energy balance (kcal/day) was calculated as follows: 1.0(kcal/g) × ⊿FFM/⊿t ＋ 9.5 (kcal/g) × ⊿FFM/⊿t, t = 221 days. The energy density of FFM was 1.0 kcal/g, and that of the FM was 9.5 kcal/g; ​​​​​​​^f^: Total energy expenditure (TEE) measured using the DLW method; ​​​​​​​^g^: TEE Japan-DRI was estimated using the following equation of DRI for the Japanese population: resting energy expenditure (REE) × physical activity coefficient (PAL) + energy deposition; ​​​​​​​^h^: TEE equation by Kaneko et al.; REE predicted by equation of Kaneko et al. ×PAL+ energy deposition; ​​​​​​​^i^: REE Japan-DRI was estimated using the DRI for Japanese individuals. REE standard (kcal/kg/day) × body weight (kg). The REE values used for boys aged 15–17 years and 12–14 years were 27 and 31; ​​​​​​​^j^: REE equation proposed by Kaneko et al. For boys: 14.4 × body weight + 5.09 × height − 34.0 × age + 403; ​​​​​​​^k^: Physical activity (PA) excluding training time was measured using an accelerometer. Moderate and vigorous physical activity (MVPA), sedentary behavior (SB), and low physical activity (LPA);​​​​​​​ ^l^: Physical activity level (PAL) was calculated as TEEDLW/REE, according to the equation used in Kaneko et al. study.

Variables	Mean ± SD
Total energy accumulation over 6 months	
Energy store of FFM (kcal/six months)^a^	2,133 ± 3,339
Energy store of FM (kcal/six months)^b^	−1,001 ± 45,167
Total energy accumulation for six months^c^	1,132 ± 42,737
Total energy accumulation/kg for six months^d^	565.7
Energy balance (kcal/day)^e^	5.6 ± 213
TEE (kcal/day)	
TEE measured^f^	4,194 ± 734
Japan-DRI^g^ (PAL 1.5)	4,262 ±754
Japan-DRI^g^ (PAL 1.75)	4,970 ± 880
Japan-DRI^g^ (PAL 2.0)	5,677 ± 1,006
Equation by Kaneko et al.^h^	4,202 ± 500
BMR (kcal/day)	
Japan-DRI^i^	2,830 ± 503
Equation by Kaneko et al.^j^	2,202 ± 263
PA (min/day)^k^	
Training time	155 ± 10
MVPA excluding training	57 ± 14
Sleeping time	499 ± 46
SB	516 ± 69
LPA	214 ± 44
PAL^l^	1.90 ± 0.19

Physical activity

**Figure 2:**
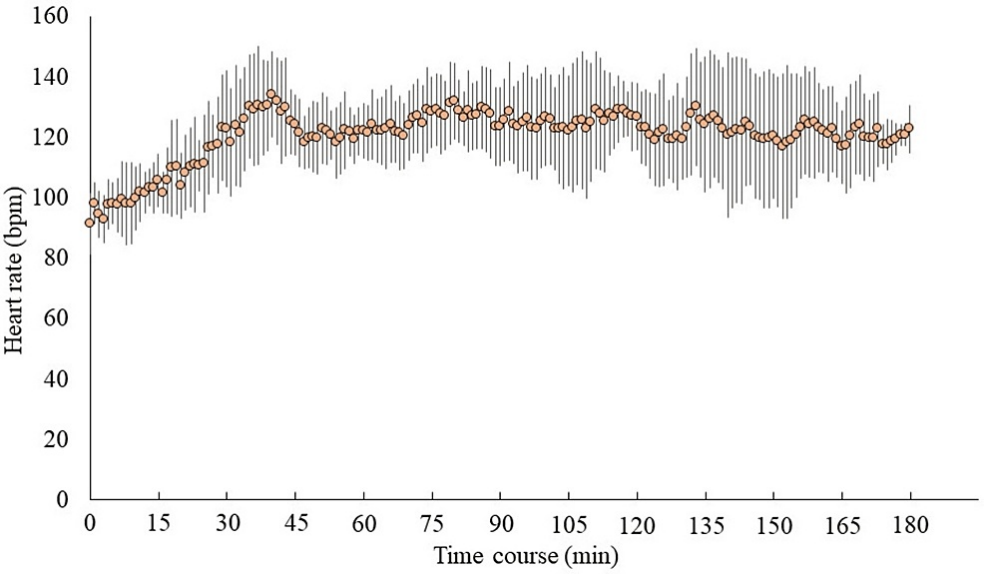
Changes in heart rate during training sessions. Average heart rate, 121 ± 9 beats/minute; maximum heart rate, 157 ± 12 beats/minute; minimum heart rate ± 81 beats/minute.

The duration of non-training MVPA was 57 ± 14 minutes/day, while the training duration was 155 ± 10 minutes/day. In daily life, SB accounted for 516 ± 69 minutes/day, sleep for 499 ± 46 minutes/day, and LPA for 214 ± 44 minutes/day outside of training. Sumo training sessions typically lasted 2.5-3 hours per day and were conducted 5-6 days per week. During training, the average HR was 121 ± 9 beats/minute, with an average maximum HR of 157 ± 12 beats/minute and an average minimum HR of 81 ± 8 beats/minute (Figure 2).

## Discussion

We used DLW and BIA methods to determine the body composition and TEE of junior Sumo wrestlers. The results indicated a significant increase in body weight, while the percentages of body fat and FM remained unchanged within six months. In addition, the FFM increased significantly. The energy balance showed an average surplus of 5.6 ± 213 kcal/day. Maintaining a certain physique could, therefore, be accomplished through a unique Sumo lifestyle characterized by predominant sedentary behavior outside of training.

Characteristics of body size and changes in body size, body composition, and energy balance within six months

The average height of junior Sumo wrestlers was comparable to Japanese males aged 15-17, recorded as 170.1 cm [15]. However, their average body weight was 1.68 times higher than that of Japanese males aged 15-17 years, recorded as 59.7 kg. This physique falls within the classification of obesity based on the BMIz and obesity assessment (Table 1) [16,17].

Stature and mass exhibited substantial increments of 0.8 cm and 2.0 kg, respectively, within six months (Figure 1). No notable changes were observed in the BMI, BMIz, or composite measure of BMI. Employing the regression equation, we adjusted the TBW estimated using BIA after six months to obtain the adjusted FFM and FM values. We subsequently compared the changes in body composition before and after six months, which revealed an FFM increase of 2.1 kg and an FM decrease of 0.1 kg, indicating that skeletal muscle and organs/tissues exhibit greater increments than adipose tissue despite being at an age where fat tends to accumulate. This can be attributed to the effects of persistent training. Subsequently, the energy stored within six months was determined based on the increments in FFM and FM.

According to the Japanese Dietary Reference Intakes (2020), energy accumulation (energy storage) values equivalent to the tissue increment required for growth in Japanese men were set at 10 kcal/day for individuals aged 15-17 years and 20 kcal/day for those aged 12-14 years. This amount of energy storage can be calculated by multiplying the yearly weight gain with the energy density of tissue increment, which is 1.9 kcal/g [15]. By converting the weight gain of junior Sumo wrestlers, the required energy accumulation amounted to 18.9 kcal/day for individuals aged 15-17 years and 14.9 kcal/day for those aged 12-14 years. In contrast, junior Sumo wrestlers’ actual daily energy balance was +5.6 ± 213 kcal. FFM increased during training periods despite a negative balance, suggesting the activation of compensatory mechanisms for the maintenance of physique [18]. In this study, despite the necessity of energy accumulation for growth, FFM increased within six months, even with a slightly positive balance. Daily physical activity should be evaluated to elucidate the compensatory mechanisms of energy balance in junior Sumo wrestlers.

Characteristics of training and physical activity of junior Sumo wrestlers

Junior Sumo wrestler training spanned an average duration of 2.5-3 hours and was conducted 5-6 times per week. The breakdown of daily physical activity among junior Sumo wrestlers is shown in Table 2. Previous studies that evaluated physical activity in children using accelerometers reported MVPA levels of 55.8 ± 18.3 minutes/day, LPA levels of 427.8 ± 53.4 minutes/day, and SB levels of 312.0 ± 56.2 minutes/day among children without obesity [19]. Unlike these figures, junior Sumo wrestlers frequently engage in MVPA because of their training; however, non-MVPAs largely comprise SB (516 ± 69 minutes/day). Sumo wrestlers develop a high proportion of FFM through continuous training and consume substantial energy to promote weight gain [1]. Professional Sumo wrestlers generally train in the morning without consuming breakfast. They typically have two meals a day, lunch and dinner, with an estimated calorie intake of approximately 5,000 kcal [20]. After lunch, they engage in minimal activity, often taking a nap until dinner, and may consume snacks or a late-night meal if required. The traditional practice of consuming energy in a fasting state followed by prolonged rest periods contributes to the body composition of professional Sumo wrestlers [21]. Junior Sumo wrestlers also adopt a similar lifestyle, limiting activities outside of training to maintain their physique.

Total energy expenditure of junior Sumo wrestlers

The estimated energy requirement for Japanese men aged 15-17 years is calculated based on age-specific basal metabolic rate (BMR), body weight, physical activity level (PAL), and energy stores. Based on that formula, the estimated energy requirements for junior Sumo wrestlers were 4,970 ± 880 kcal/day when the PAL was set at a normal value of 1.75 and 5,677 ± 1,006 kcal/day when the PAL was set at 2.0 due to daily training [15]. The actual TEE measured using DLW of 4,194 ± 734 kcal/day was lower than the estimated value. This discrepancy may be attributed to the fact that body composition was not considered when determining the BMR in the estimated energy requirement. BMR estimates based on the Dietary Reference Intakes for Japanese individuals were calculated as follows: BMR was obtained by multiplying body weight by 27 kcal/day and 31 kcal/day for individuals aged 15-17 years and 12-14 years, respectively [15]. Consequently, the BMR adjusted for body size was 2,830 ± 503 kcal/day.

**Figure 3:**
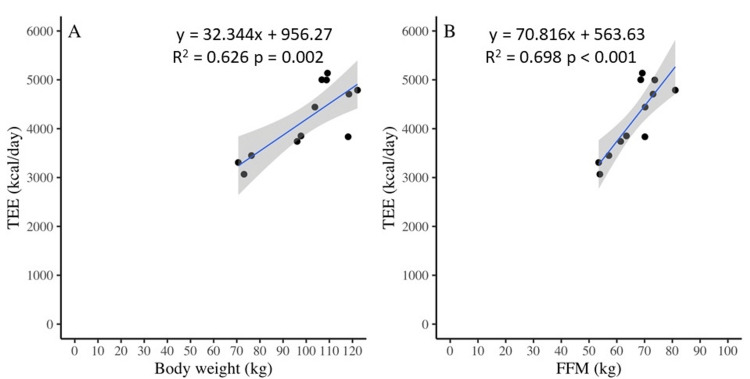
Relationship among total energy expenditure (TEE), body weight, and fat-free mass (FFM).

Kaneko et al. reported a strong correlation between BMR and FFM in Japanese individuals aged 6-17 years. According to Kaneko et al.’s formula, which incorporates body composition, the BMR was 2,202 ± 263 kcal/day [22]. We examined the relationship between TEE, FFM, and body weight in junior Sumo wrestlers and found that the correlation between TEE and FFM was stronger than that between TEE and body weight. These findings suggest that body composition should be considered in obese and overweight children (Figure 3).

During the growth and development phase in adolescents, the need for energy to support the growth of organs and tissues increases, resulting in a higher metabolic rate than in adults [23]. Therefore, energy requirements should be calculated by considering body composition and PALs. The measured TEE values of junior Sumo wrestlers were close to the estimated values of 4,202 ± 500 kcal/day obtained using Kaneko et al.’s formula, which considers body composition.

If the body weight remains unchanged during the measurement period, TEE can be estimated as energy intake using the DLW method [24]. The estimated energy intake of college students and professional Sumo wrestlers reportedly ranges from 3,098 to 5,586 kcal/day [25]. In this study, when the TEE of the Sumo group was considered to be 4,190 ± 734 kcal/day, the energy intake during the same period was similar to that of college students and professional Sumo wrestlers. However, only a slight positive balance was observed when energy requirements for growth were considered.

Application to children with obesity

A persistent positive energy balance leads to weight gain, particularly excessive adipose tissue accumulation [26-28]. Childhood obesity increases the risk of lifestyle-related diseases [29,30]. Despite consuming less than the estimated energy requirement, junior Sumo wrestlers experience weight gain. Furthermore, although SB is commonly associated with obesity [19], junior Sumo athletes engage in 2-3 hours of MVPA per day but are sedentary outside of training. This suggests that a sedentary lifestyle serves as a compensatory mechanism for intentionally increasing body weight, even with a small surplus of energy. A high TEE does not guarantee weight loss, and a low TEE does not necessarily lead to obesity, particularly in children [28]. A persistent positive energy balance is expected in children with obesity who consume the same amount of calories as junior wrestlers but lack exercise, even after accounting for the energy requirements for growth. The absence of exercise promotes SB, increasing the propensity for obesity [19].

Limitations

In this study, notable interindividual variations were observed in TEE and energy balance. Junior Sumo athletes undergo a period of growth and development, and their energy requirements for growth and susceptibility to fat accumulation may differ with age. This study included 12 male Sumo wrestlers. The limited sample size of male Sumo wrestlers is due to the practicality and cost constraints of this study. Although the study’s sample size was limited, conducting a more comprehensive analysis of the results within specific age groups could have provided clearer insights into the impact of growth and development processes. Future studies should include long-term longitudinal observations. Moreover, a year-long investigation is necessary to consider seasonal variations and the influence of competition and training camps on individual weight changes. Therefore, it is necessary to investigate the dietary intake of junior sumo wrestlers. In this study, DLW was not analyzed in the control group, partly because it is an expensive measurement and should be performed in future studies.

## Conclusions

Although the height and weight of junior Sumo athletes increased significantly throughout the six months, an increase of 2.1 kg in FFM and a reduction of 0.1 kg in FM were observed. Persistent training yielded more pronounced augmentation in FFM than in FM. The measured TEE was 4,194 ± 734 kcal/day, resulting in a slightly positive energy balance within six months. It has been postulated that compensatory mechanisms similar to those observed in adult athletes may be involved, with their distinct lifestyle, characterized by low activity levels outside training. To facilitate healthy competition, nutritional guidance and regular medical checkups should be offered to overweight junior athletes. For instance, in practice, the TEE data from this study could be used as a reference for nutritional guidance when calculating the energy requirements of junior Sumo wrestlers.
